# Acute leukemia cells resistant to PI3K/mTOR inhibition display upregulation of P2RY14 expression

**DOI:** 10.1186/s13148-018-0516-x

**Published:** 2018-06-19

**Authors:** Kinjal Shah, Sausan A. Moharram, Julhash U. Kazi

**Affiliations:** Division of Translational Cancer Research, Department of Laboratory Medicine, Lund University, Lund, Sweden

**Keywords:** AML, ALL, B-ALL, P2RY14

## Abstract

The PI3K/mTOR pathway is the second most frequently deregulated pathway in a majority of cancers such as breast cancer, lung cancer, and melanomas as well as leukemia. Mutations in the genes coding for receptor tyrosine kinases (RTKs) and G-protein-coupled receptors (GPCRs) are quite common in all forms of acute leukemia. This can be a major cause of deregulation of the PI3K-mTOR pathway. To understand how cells display resistance to the dual PI3K/mTOR inhibitor, we used a panel of 25 acute leukemia cell lines. We observed that while a number of cell lines displayed sensitivity to the dual PI3K/mTOR pathway inhibitor PKI-587, many cells displayed substantial resistance. Cells sensitive to PKI-587 also showed aberrant activation of PI3K/mTOR pathway components such as AKT and S6K and also displayed sensitivity to a panel of various other PI3K/mTOR inhibitors. Using RNA sequencing data, we observed that expression of a G protein-coupled receptor, P2RY14, was upregulated nine-fold in cells showing resistance to the PI3K/mTOR inhibitor. P2RY14 has not been much studied in hematologic malignancies. However, this receptor seems to have a role in the localization of hematopoietic stem cells (HSCs) and in promoting regenerative capabilities following injury. We observed that acute lymphoblastic leukemia (ALL) and FLT3-ITD-positive acute myeloid leukemia (AML) patients with higher expression of P2RY14 mRNA displayed relatively poor survival compared to patients carrying lower expression of P2RY14 suggesting a role of P2RY14 in patient survival. To understand the role of this receptor in cell signaling, we used phospho-protein arrays and observed activation of distinct signaling cascades. Furthermore, array data were verified using murine pro-B cell line Ba/F3 stably transfected with P2RY14. We observed that activation of P2RY14 by its ligand, UDP-glucose, resulted in selective induction of ERK1/2 phosphorylation. Taken together, our data suggest that acute leukemia cells resistant to PI3K/mTOR inhibition display upregulation of a GPCR, P2RY14, which has a role in patient survival and also couples to the activation of ERK signaling.

**Electronic supplementary material:**

The online version of this article (10.1186/s13148-018-0516-x) contains supplementary material, which is available to authorized users.

The success of targeted therapy relies on carefully identifying and targeting specific genes or molecules of a particular signaling pathway. One such central signaling center of cancer development is the phosphoinositide 3-kinase-AKT-mammalian target of rapamycin (PI3K/AKT/mTOR) pathway which plays a key role in cell survival, growth, and metabolism. This pathway is highly deregulated and hyper-activated in different cancers due to the high frequency of mutations observed in its various components, and thus, there is tremendous interest in inhibiting elements of this pathway [1]. Although the loss of PTEN is one of the common mutations that activates the PI3K/mTOR pathway, mutations in upstream receptors, such as receptor tyrosnie kinases (RTKs) and G-protein-coupled receptors (GPCRs), also contribute. Several drugs targeting this pathway have been developed that could prevent uncontrolled proliferation of cells [2]. However, many of these agents have not been fully efficient in their action, in either the preclinical or clinical setting, which demands more research into effectively targeting this pathway [2–4].

We have recently shown that the dual PI3K/mTOR inhibitor PKI-587 can inhibit the growth of acute myeloid leukemia (AML) as well as acute lymphoblastic leukemia (ALL) cell lines both in vitro and in vivo [3, 4]. However, the effect on in vivo growth inhibition was marginal. To understand why cells display poor response to PKI-587, we used a panel of 25 acute leukemia cell lines. We first characterized cell lines with respect to drug response and activation of the PI3K/mTOR pathway. We observed that while some cell lines were highly sensitive, others displayed resistance to PKI-587 (Additional file 1: Table S1). Additionally, we observed that cells which responded to PKI-587 displayed constitutive activation of PI3K/mTOR pathway components (Fig. 1a and Additional file 1: Figure S1) suggesting that cells require activation of the PI3K/mTOR pathway in order to respond to PKI-587. This combined approach allowed us to generate two sets of cell lines as “PKI-587 sensitive” and “PKI-587 resistant” where each group contains ten cell lines. This classification was further supported by data from the COSMIC database where we observed that a panel of inhibitors targeting the PI3K/mTOR pathway also displayed efficacy against PKI-587-sensitive cells, whereas the other group showed substantial resistance (Additional file 1: Table S2). Use of additional PI3K/mTOR inhibitors, Apitolisib, BGT226, and Dactolisib, showed similar response that we observed for PKI-587 (Fig. 1b). Furthermore, there was an enrichment of kinase and metabolic signaling pathways in sensitive cells (Additional file 1: Figure S2A), while resistant cells displayed enrichment of genes regulated by transcription factors (Additional file 1: Figure S2B) further supporting the data that sensitive cells are mainly dependent on the PI3K/mTOR pathway. Activation of the PI3K/mTOR pathway can be mediated through different mechanisms in acute leukemia. For example, around 50% of T-cell acute lymphoblastic leukemia (T-ALL) patients have mutations in the NOTCH1 receptor leading to inhibition of PTEN which thereby activates AKT and promotes resistance to glucocorticoid therapy, an important indicator of therapeutic failure in T-ALL [5, 6]. However, we have not seen any common mutational pattern neither in sensitive nor in resistant cell lines (data not shown).

**Fig. 1:**
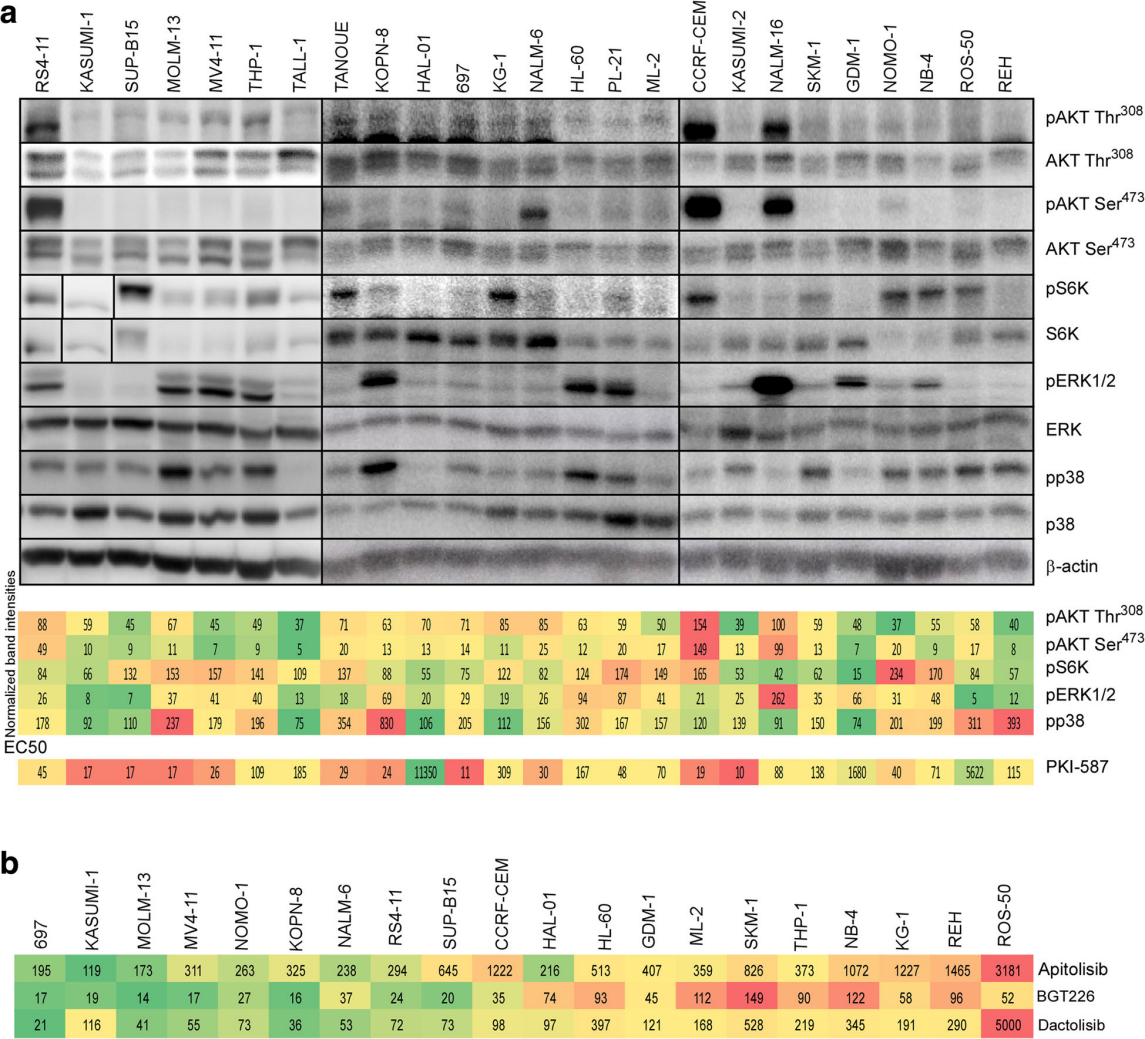
PI3K/mTOR pathway enrichment and drug sensitivity. **a** Around 25 acute leukemia cell lines were checked for their basal signaling of AKT, ERK, S6K, and p38 pathways after starving them for 4 h. The heatmap shows the average quantification of phosphorylated proteins along with the average EC_50_ values of PKI-587. **b** The group of 20 cell lines that showed sensitivity (697, KASUMI-1, MOLM-13, MV4-11, NOMO-1, KOPN-8, NALM-6, RS4;11, SUP-B15 and CCRF-CEM) or resistance (HAL-01, HL-60, GDM-1, ML-2, SKM-1, THP-1, NB-4, KG-1, REH and ROS-50) to PKI-587 were further verified with three PI3K/mTOR inhibitors Apitolisib, BGT226, and Dactolisib

To identify the major regulators of the resistant phenotype, we compared gene expression between the PKI-587-sensitive and PKI-587-resistant cell lines. Several genes were found to be upregulated or downregulated in resistant cells in comparison to the sensitive cells (Fig. 2a). A GPCR, P2RY14 (also known as GPR105), was upregulated nine-fold in the set of resistant cell lines (Fig. 2b). Upregulation of P2RY14 mRNA expression was further verified by RT-qPCR (Fig. 2c). P2RY14 has so far not been thoroughly studied in connection to cancer. One report suggests that activation of P2RY14 through its ligand, UDP-glucose, results in the induction of Tyr705 phosphorylation of signal transducer and activator of transcription 3 (STAT3) in epidermal keratinocyte cell line HaCaT [7]. We observed that in patients with ALL as well as in oncogenic FLT3-ITD-positive AML, P2RY14 expression correlated with overall survival where patients carrying the higher expression of P2RY14 had a relatively poor survival (Fig. 2d).

**Fig. 2:**
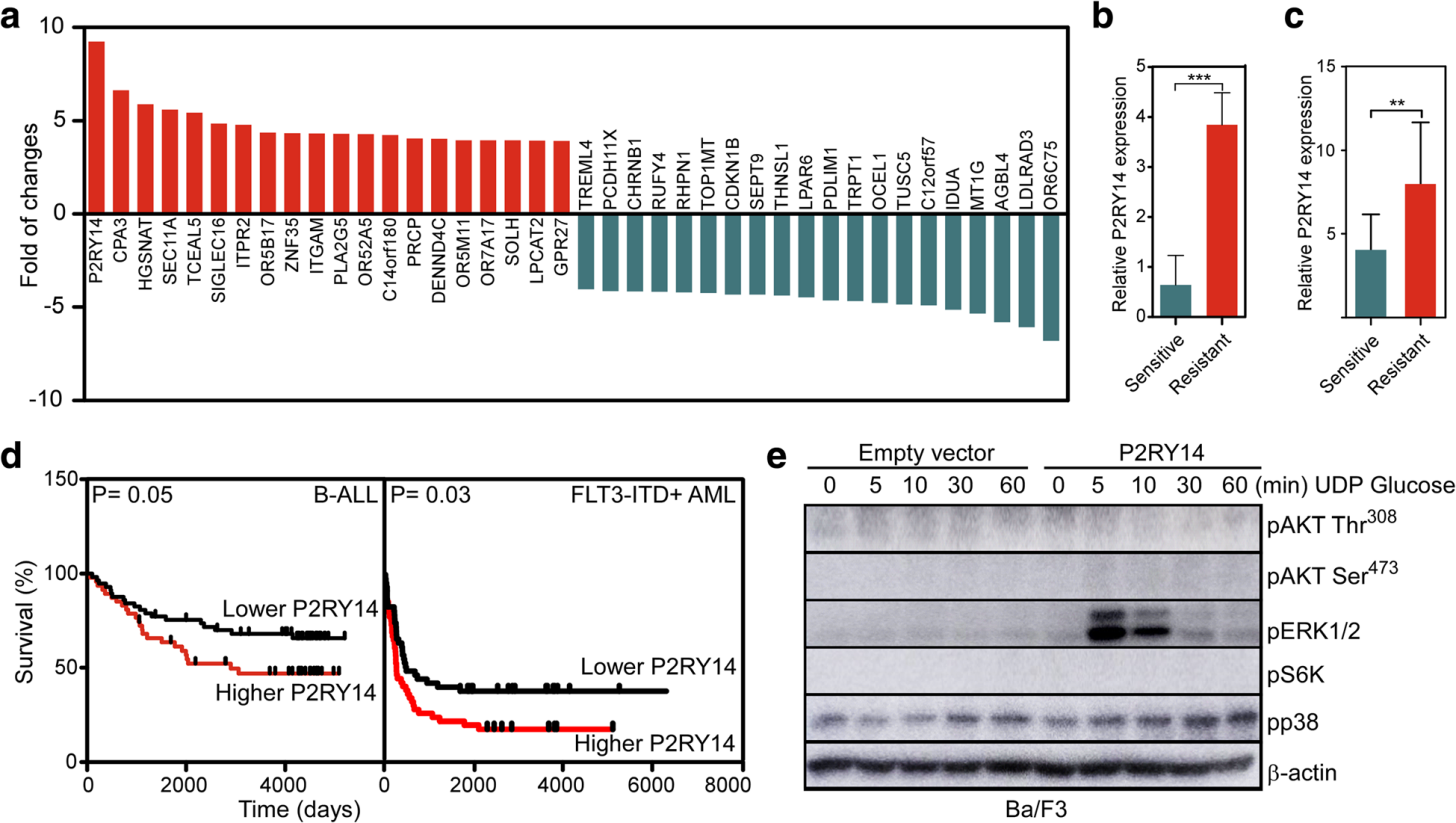
RNA sequencing data were used to analyze gene expression in PKI-587-sensitive and PKI-587-resistant sets of cell lines. **a** Upregulated and downregulated genes (top 20 each) in resistant cells as compared to sensitive cells. **b** Relative P2RY14 expression from RNAseq data. **c** Relative P2RY14 expression from RT-qPCR data. **d** Kaplan-Meier survival plot shows the overall survival benefit of ALL and AML patients. **e** Western blot was performed for the total cell lysate to determine phosphorylation of AKT, ERK1/2, S6K, and p38

RTKs and GPCRs, which couple to different G proteins, such as G_i/o_, G_s_, and G_q_, can activate ERK1/2 which are kinases belonging to the mitogen-activated protein kinase (MAPK) family [8]. P2RY14 is a G_i_-coupled receptor which is activated by UDP-glucose. ERK, JNK, and p38 MAPKs seem to be activated upon stimulation of G_i_-coupled receptors with UDP-glucose along with mobilization of intracellular Ca^2+^ stores [7]. In order to understand the signaling pathways regulated by P2RY14, we stimulated cells with 100 μM UDP-glucose and analyzed activation of receptor and non-receptor tyrosine kinases using Proteome Profiler Human Phospho-RTK Array (ARY001B) and Proteome Profiler Human Phospho-Kinase Array (ARY003B) Kits from R&D Systems. We did not see any activation of receptor tyrosine kinases in response to UDP-glucose (data not shown). However, there was a significant upregulation of phosphorylation of AMPKa2, c-JUN, ERK1/2, FAK, GSK-3alpha/beta, JNK, p53, PRAS40, SRC, and STAT5a (Additional file 1: Figure S3).

To further explore the signaling downstream of P2RY14, we used the murine pro-B cell line Ba/F3. We stably transfected Ba/F3 cells with *P2RY14* or empty vector using the retroviral system. P2RY14 expression in Ba/F3 cells was determined by flow cytometry (Additional file 1: Figure S4A) and Western blotting (Additional file 1: Figure S4B). We starved cells of serum and cytokines and stimulated with 100 μM UDP-glucose for different periods of time. We did not see any phosphorylation of AKT and S6K in response to UDP-glucose stimulation suggesting that PI3K/mTOR signaling is not occurring downstream of P2RY14 (Fig. 2e). Similar to PI3K/mTOR signaling, p38 signaling was also not activated. However, we observed strong activation of ERK signaling only in Ba/F3 cells expressing P2RY14 (Fig. 2e) indicating that Ba/F3 cells do not express P2RY14 or the level of expression is extremely low. ERK phosphorylation decreased exponentially over the time (Additional file 1: Figure S5), demonstrating that ERK activation by P2RY14 is transient. This is in line with earlier observation [9] where a transient increase in ERK1/2 phosphorylation was observed in cells stimulated with UDP-glucose with peak activation occurring at 5 min. Thus, these data suggest that P2RY14 may couple to ERK signaling in lymphocytic cells.

P2RY14 is widely expressed in the placenta, adipose tissue, intestine, and stomach whereas it is moderately expressed in the brain, spleen, liver, and lung [7]. It is also selectively expressed in subpopulations of bone marrow hematopoietic stem cells (HSCs) where they might play a role in bone marrow cell localization and compartmentalization as well as to promote regenerative responses after injury. Moreover, increased senescence of HSCs was observed in P2RY14 knockout mice in response to aging, chemotherapy, radiation, and other environmental stresses [10]. With such important roles of P2RY14 in lymphocytes, further investigation into the activation of this receptor by UDP-glucose is definitely required in terms of additional signaling such as JNK and STAT as well as measuring the intracellular concentration of Ca^2+^ and cAMP. By constitutive release from certain physiologically relevant tissues as well as release during tissue injury and inflammation, UDP-glucose can serve as an autocrine or paracrine activator of P2RY14, thereby inducing the expression of IL-8, a mediator of inflammation [7]. Thus, the release of UDP-glucose from lymphocytes also needs to be investigated. ERK can phosphorylate and activate certain transcription factors which lead to cellular proliferation [7]. Since ERK signaling is activated upon P2RY14 stimulation by UDP-glucose, it can promote cellular growth. Thus, it would be interesting to check the downstream signaling effects of P2RY14 inhibition by antagonists. Further, inhibiting MAPK along with PI3K/AKT/mTOR can serve as an effective combination for anti-leukemic therapy since both are major pro-survival and anti-apoptotic pathways. However, secondary drug resistance is a major drawback of targeted therapy which needs to be tactfully handled by regulating various feedback loops [5]. Future work would thus include various in vitro assays to check the functionality of P2RY14 and later on in vivo preclinical trials to determine the effect of overexpression as well as inhibition of P2RY14 on the physiology and metabolism of mice.

## Additional file

Additional file 1: Additional file includes supplementary materials and methods, supplementary tables S1-S2 and supplementary figures S1-S5. (PDF 997 kb)

## Abbreviations

ALL: Acute lymphoblastic leukemia
AML: Acute myeloid leukemia
GPCR: G-protein-coupled receptor
HSC: Hematopoietic stem cell
MAPK: Mitogen-activated protein kinase
mTOR: Mammalian target of rapamycin
PI3K: Phosphoinositide 3-kinase
RTK: Receptor tyrosine kinase
STAT3: Signal transducer and activator of transcription 3
T-ALL: T cell acute lymphoblastic leukemia
