# Supplementary material for: Acute leukemia cells resistant to PI3K/mTOR inhibition display upregulation of P2RY14 expression
Source: Clin Epigenetics. 2018 Jun 19;10:83. doi: 10.1186/s13148-018-0516-x (PMC6010022; doi:10.1186/s13148-018-0516-x)

Acute leukemia cells resistant to PI3K/mTOR inhibition display upregulation of P2RY14 expression

Kinjal Shah, Sausan A. Moharram and Julhash U. Kazi

Division of Translational Cancer Research, Department of Laboratory Medicine, Lund University, Lund, Sweden

Supplementary materials and methods

Antibodies:
Different types of antibodies were used for immunodetection of proteins which are mentioned in table 1 along with their dilutions.

Table 1: Different antibodies used along with their dilutions

Antibodies and their dilutions used	Company
Rabbit anti-phospho-ERK1/2 (pThr202/pThr204) (1:200),<br>Goat anti-AKT1/2 (1:200)<br>Horseradish peroxidase-coupled secondary anti-goat antibody (1:100,000)<br>Horseradish peroxidase-coupled β-actin (1:5000)	Santa Cruz Biotechnology
Rabbit anti-phospho AKT (pThr308) (1:5000)	Epitomics
Rabbit anti-phospho AKT (pSer473) (1:5000),<br>Rabbit anti-phospho S6K (pThr389) (1:1000),<br>Rabbit anti-S6K (1:1000)	Abcam
Mouse anti-phospho-p38 (1:2500), Mouse anti-p38 (1:5000)	BD Biosciences
Rabbit anti-ERK2 (1:200)	Homemade
Horseradish peroxidase-coupled secondary anti-rabbit and anti-mouse antibodies (1:100,000)	ThermoFisher Scientific
Alexa Fluor 647 anti-FLAG (1:200),<br>Horseradish peroxidase-coupled anti-FLAG (1:2000)	Sigma-Aldrich

Plasmids and expression constructs:
Mouse purinergic receptor P2Y, G-protein coupled, 14 (*P2RY14*), transcript variant 1 in the pCMV6-Myc-DKK vector was obtained from Origene. This plasmid was amplified in *E.coli* DH5α in the presence of 25µg/ml Kanamycin. Sub-cloning was performed by transferring the open reading frame (ORF) of *P2RY14* gene with Myc-DKK tag from its original vector (pCMV6) to the pMSCVneo vector. The final expression plasmid construct so generated, pMSCVneo-*P2RY14*-Myc-DKK was then amplified in *E.coli* DH5α supplemented with 100µg/ml Ampicillin and these were used for retroviral transfection of Ba/F3 cells.

Cell lines and media:
All the different types of acute leukemia cell lines used in the project as mentioned in table 2 were cultured in their respective media according to table 3. Ecopack cells (packaging cell line for the viral particles) were cultured in DMEM supplemented with 10% FBS and 1% Penicillin-Streptomycin. On the other hand, Ba/F3 cells (murine pro B cells) were maintained in RPMI1640 supplemented with 10% FBS, 1% Penicillin-Streptomycin and 10 ng/ml recombinant murine interleukin3 (IL-3). All the cell lines used were obtained from DSMZ, Germany and maintained in Heraeus BBD 6220 Incubator (ThermoFisher Scientific, USA) at 37°C with 5% CO_2.

Table 2: Different types of leukemia cell lines used

Leukemia type	Cell lines
T cell leukemia	CCRF-CEM, TALL-1
B cell leukemia	RS411, SUP-B15, ROS-50, 697, TANOUE, KASUMI-2, KOPN8, HAL-01, NALM6, NALM16, REH
Acute myeloid leukemia/ Acute monocytic leukemia	KASUMI-1, NOMO-1, MOLM13, MV411, THP-1, NB4, HL60, PL-21, SKM-1, GDM-1, ML-2, KG-1

Table 3: Different types of media used for culturing leukemia cell lines

Media used	Cell lines
90% RPMI1640 + 10% FBS + 1% Penicillin-Streptomycin	RS411, CCRF-CEM, NOMO-1, MOLM13, MV411, THP-1, NB4, HL60, KG-1, TANOUE, KASUMI-2, KOPN8, NALM16, HAL-01, REH, NALM6
80% RPMI1640 + 20% FBS + 1% Penicillin-Streptomycin	KASUMI-1, TALL-1, PL-21, SKM-1, GDM-1, ML-2, ROS-50, 697
80% McCoy's 5A + 20% FBS + 1% Penicillin-Streptomycin	SUP-B15

Immunoprecipitation and Western Blot:
Cells were washed thrice with 1X DPBS or 1X RPMI1640 medium to remove serum and cytokines if any. The cells were then re-suspended in 1X RPMI1640 medium and kept for 4 hours at 37°C with 5% CO_2 thus starving them of serum and cytokines. After incubation, Ba/F3 cells stably transfected with *P2RY14* and Empty vector were stimulated with 100µM UDP-glucose at 37°C for the respective amounts of time. The stimulation was stopped by immediately placing the cells on ice and adding ice cold 1X PBS. Thereafter, these cells, as well as the leukemia cell lines, were lysed in the same manner in 1% Triton X-100 lysis buffer supplemented with 3 protease inhibitors (1mM Na3VO4, 1% Trasylol and 1mM PMSF) and incubated for 15 minutes on ice. Immunoprecipitation was performed using 1µg primary antibody for 1 ml cell lysates followed by incubation for 1 hour on ice. Immunoprecipitates were collected using magnetic beads which were washed thrice with the above-mentioned lysis buffer containing the protease inhibitors. The lysates were mixed with 1:1 volume of SDS sample loading buffer and boiled at 95°C for 5 minutes. The protein concentration of the total cell lysates was determined by BCA Protein Assay Kit (ThermoScientific, USA) and 20µg proteins from each lysate were separated on 8.5% SDS-PAGE gels followed by their transfer to PVDF membranes. These membranes were then blocked with 5% non-fat dry milk prepared in 0.2% PBS-T for 1 hour at room temperature. Prior to incubation with the respective primary antibodies overnight at 4°C, the membranes were washed thrice in 0.05% PBS-T for 5 minutes each. For immunodetection, the blots were incubated with the respective horseradish peroxidase-conjugated secondary antibodies for 1 hour at room temperature and were developed with the Luminata Forte Western HRP Substrate (Millipore) and Amersham Imager 600 (GE Healthcare, Sweden). The band intensities were quantified with the Multi- Gauge software from Fujifilm.

Inhibitor Assay/ Cell Viability Assay:
All the above mentioned 25 acute leukemia cell lines were tested for their viability against 10 different concentrations of the dual PI3K-mTOR inhibitor Gedatolisib (PF-05212384, PKI-587) ranging from 0.1nM to 5000nM. To perform this assay, all the cell lines were seeded at a density of 15,000 cells/well in a 96-well plate. Medium blank and cell blank (cells with no inhibitor) were used as controls. About 10µl Gedatolisib was added to 15,000 cells present in 90µl per well. Thus, each column in a 96-well plate represents one concentration of the inhibitor. The plate with the inhibitor was then incubated at 37°C - 5% CO_2 for 48 hours. After the incubation period, cell viability was determined by adding 10µl PrestoBlue in each well and then measuring the fluorescence with a 96-well plate reader after 2-5 hours depending on the color change from blue to purple in the cell without inhibitor column. EC50 values (nM) were determined using GraphPad Prism software and thus, the

relative viability curves were generated. Later three more inhibitors Apitolisib, BGT226 and Dactolisib were used to measure EC50 values using the same method.

Stable transfection of Ba/F3 cells:

The expression plasmid constructs pMSCVneo-*P2RY14*-Myc-DKK and pMSCVneo-empty vector were used for retroviral transfection of Ba/F3 cells. To achieve this, the packaging cell line for viral particles, EcoPack was transfected with both the vectors using Lipofectamine 2000. After incubating the transfected cells for 72 hours, the supernatant containing the viral particles was collected and were then used to infect Ba/F3 cells. Both the stably transfected Ba/F3 cells were selected for a period of 2 weeks against 0.8 mg/ml geneticin (G-418), an analog of neomycin. Flow cytometry and Western Blot were used to confirm the expression of P2RY14 using anti-Flag antibodies.

RNAseq, mutation and inhibitor data from COSMIC Database:

COSMIC (Catalogue of Somatic Mutations In Cancer) is the world's largest database for containing the most comprehensive set of data for exploring the impact of somatic mutations in various human cancer cell lines. Expression Analysis in the form of RNA Sequence Data was downloaded from COSMIC for both PKI-587 sensitive and resistant sets of cell lines used in this project. This dataset was used to calculate the p-value and fold of expression changes for 16,445 genes in these 2 sets of cell lines. Inhibitor Data which gives a range of different inhibitors and their IC50 values against different cell lines was downloaded for our sensitive and resistant sets. Cancer Gene Census in COSMIC gives information about mutations in 616 genes and their implications in various cancers. Different genes mutated in our set of cell lines were identified from this Mutational Data downloaded from COSMIC.

Gene Set Enrichment Analysis (GSEA):

Gene Expression Data (RNA-Seq Data) downloaded from COSMIC Database was used to run Gene Set Enrichment Analysis (GSEA) using the GSEA software (v2.2.4) from Broad Institute with 1000 permutations to identify different pathways enriched by particular sets of genes in both PKI-587 sensitive and resistant groups of cell lines.

Survival analysis:

Gene expression data for acute lymphoblastic leukemia patients was obtained from TARGET database of National Cancer Institute (https://ocg.cancer.gov/programs/target/data-matrix). Within 207 patients 122 patient data had survival information. P2RY14 expression data from 122 patient samples were to calculate the Z-score. The Z-score 0.5 cut-off difference was used to divide two groups. Overall survival data was used to calculate the survival benefit for higher (n=47) and lower (n=57) P2RY14 gene expressing patients. Furthermore, gene expression data for acute myeloid leukemia patients carrying oncogenic FLT3-ITD mutations (GSE14468) was analyzed using similar Z-score cut-off.

Real-time Quantitative PCR (RT-qPCR):

Total RNA was isolated from cells using the RNeasy mini kit (Qiagen) following manufacturer's protocol. cDNAs were synthesized with High-Capacity cDNA Reverse Transcription Kit (Thermo Fisher Scientific Catalog number: 4368814) according to the manufacturer's directions. Gene expression was assessed by RT-qPCR using an Applied Biosystems QuantStiduo 7 Flex detection system and gene-specific RT-qPCR primer assays (Thermo Fisher Scientific). β-Actin and GAPDH were used as endogenous controls to normalize the expression data. Each sample was analyzed in quadruplicate. The comparative Ct method was used to calculate the relative changes in gene expression.

Statistical Analysis:

GraphPad Prism 5.0 software was used to perform statistical analysis where data were expressed as mean ± SE. Where applicable, we used unpaired Student's t-test and one-way ANOVA with Bonferroni's post-test. $P \leq 0.05$ was considered as significant.

Supplementary tables

Table S1: Inhibitor assay for Gedatolisib (PKI-587): Around 25 acute leukemia cell lines were tested for their sensitivity and viability against the dual PI3K-mTOR inhibitor, Gedatolisib (PKI-587). This assay was performed twice and the numbers 1st and 2nd denote the EC50 values obtained for the 1st and 2nd time respectively. Red-yellow-green is the color coding scheme used in the column of average EC50 (nM) which represents the values as low-middle-high. Thus, low and high EC50 values denote highly sensitive and resistant cells respectively.

Serial No.	Cell lines	EC50 (nM) 1st	EC50 (nM) 2nd	Average EC50 (nM)
1	RS411	43	46	45
2	KASUMI-1	19	15	17
3	SUP-B15	12	22	17
4	MOLM13	20	14	17
5	MV411	24	27	26
6	THP-1	97	120	109
7	TALL-1	201	169	185
8	TANOUE	32	27	29
9	KOPN8	24	24	24
10	HAL-01	11376	11324	11350
11	697	12	11	11
12	KG1	290	327	309
13	NALM6	31	28	30
14	HL-60	139	195	167
15	PL-21	38	58	48
16	ML-2	68	72	70
17	CCRF-CEM	21	16	19
18	KASUMI-2	10	10	10
19	NALM16	91	85	88
20	SKM-1	138	138	138
21	GDM-1	1919	1442	1680
22	NOMO-1	33	47	40
23	NB4	81	60	71
24	ROS-50	5395	5848	5622
25	REH	114	115	115

Table S2. Response to the PI3K/mTOR pathway inhibitors: Average drug response for PKI-587 sensitive and resistant cells. IC50 values were curated from COSMIC drug sensitivity database.

	DRUG_NAME	SYNONYMS	TARGET	TARGET_PATHWAY	Sen-nM	Res-nM	Fold (Res/Sen)
1	BX-912	KIN001-175	PDK1 (PDPK1)	PI3K signaling	155.80076	1905.54926	12.23068001
2	Temsirolimus	CCI-779	mTOR	TOR signaling	32.536595	294.027883	9.036836268
3	Rapamycin	AY-22989,Sirolimus,W\	mTOR	TOR signaling	32.486176	207.70865	6.393755072
4	A-443654	KIN001-139	AKT1, AKT2, AKT3	PI3K signaling	189.5634	1108.10491	5.845563462
5	GSK690693		AKT	PI3K signaling	1746.7195	9321.47343	5.336560061
6	KIN001-244		PDK1 (PDPK1)	PI3K signaling	1573.6474	8396.20511	5.335506083
7	MK-2206		AKT1, AKT2	PI3K signaling	997.74637	5156.84795	5.168495842
8	JW-7-52-1		mTOR	TOR signaling	72.959578	332.565861	4.558220709
9	BEZ235	NVP-BEZ235	PI3K (class 1), mTORC1/2	PI3K signaling	28.594851	113.654759	3.974658154
10	AZD8055	AZD8055	mTORC1/2	TOR signaling	161.18375	570.206887	3.537620142
11	KIN001-102		AKT1	PI3K signaling	662.2297	1970.21955	2.97513014
12	AZD6482	KIN001-193	PI3K beta (P3C2B)	PI3K signaling	2539.2822	6100.5417	2.402467039
13	AKT inhibitor VIII		AKT1, AKT2, AKT3	PI3K signaling	5085.9683	12100.8966	2.379270958
14	AZD6482	WO2009093972	PI3K beta (P3C2B)	PI3K signaling	2707.9185	5931.46391	2.190414451
15	CAL-101		PI3K delta	PI3K signaling	6180.9489	13319.7693	2.154971592
16	OSU-03012	AR-12	PDK1 (PDPK1)	PI3K signaling	2040.0961	4269.04931	2.092572683
17	ZSTK474	KIN001-167	PI3K	PI3K signaling	152.67729	304.104348	1.991811242
18	PIK-93		PI4K,PI3K	PI3K signaling	552.99076	938.067289	1.696352545
19	OSI-027	A-1065	mTORC1/2	TOR signaling	112.18742	184.805035	1.647288345
20	PI-103		PI3K alpha,DNAPK	PI3K signaling	79.229385	125.243	1.580764515
21	GDC0941		PI3K (class 1)	PI3K signaling	589.34548	843.694123	1.431578164
22	PF-4708671		p70 S6KA	TOR signaling	22837.552	31815.8256	1.39313645
23	GDC0941		PI3K	PI3K signaling	1060.4879	1468.86062	1.385080007
24	TGX221		PI3K beta	PI3K signaling	27303.1	37533.756	1.374706742
25	GSK2126458	EX-8678	PI3K, mTOR	PI3K signaling	2.7119163	3.69924457	1.364070332

Supplementary figures

Figure S1. PI3K/mTOR pathway activation: Intensity of multiple blots has been quantified and normalized.

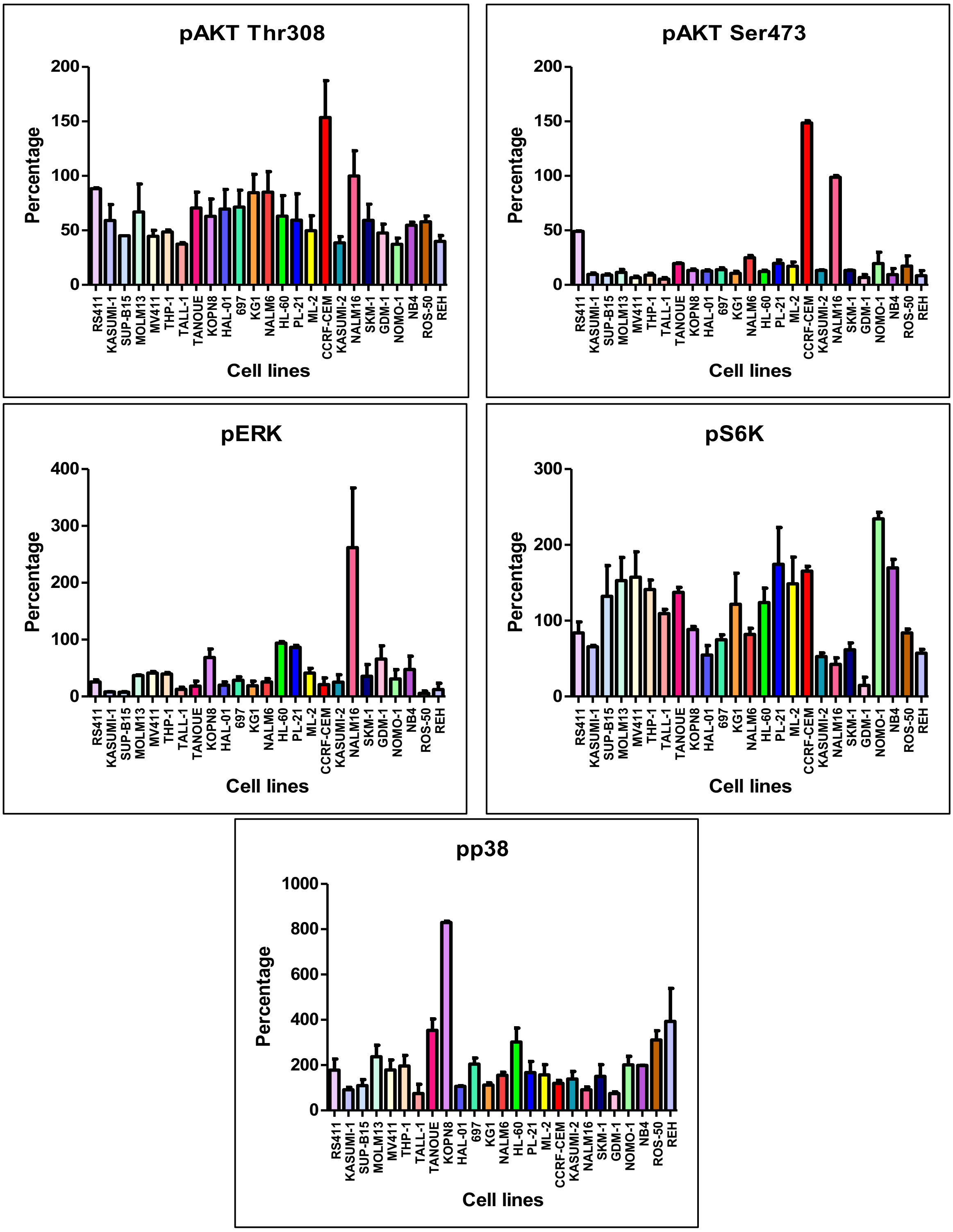

Figure S2: Gene set enrichment analysis using gene expression data from the COSMIC database: Different pathways enriched in sensitive cells (A) and resistant cells (B) were identified by Gene Set Enrichment Analysis (GSEA) using the GSEA software (v2.2.4) from the Broad Institute.

A.

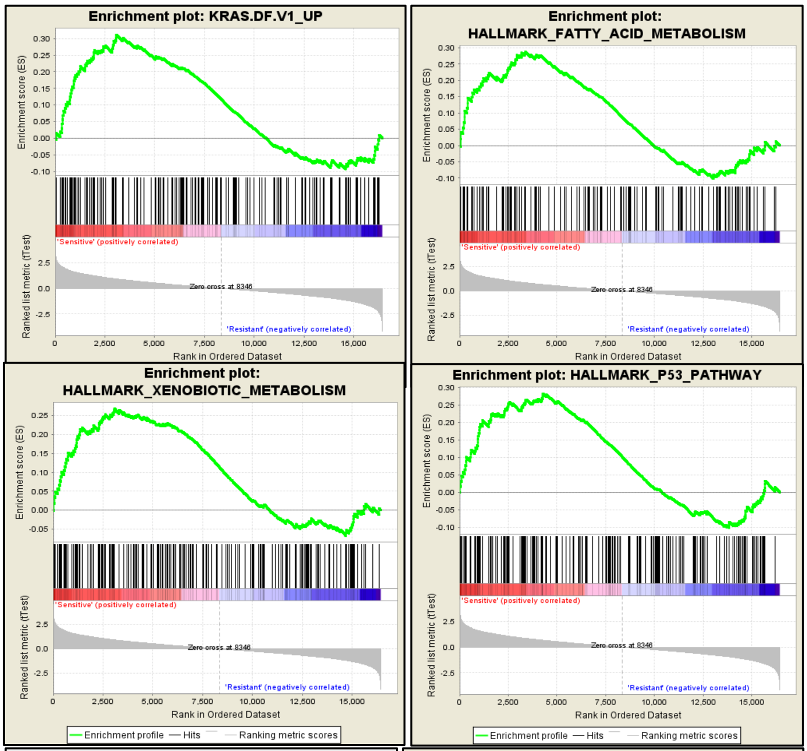

B.

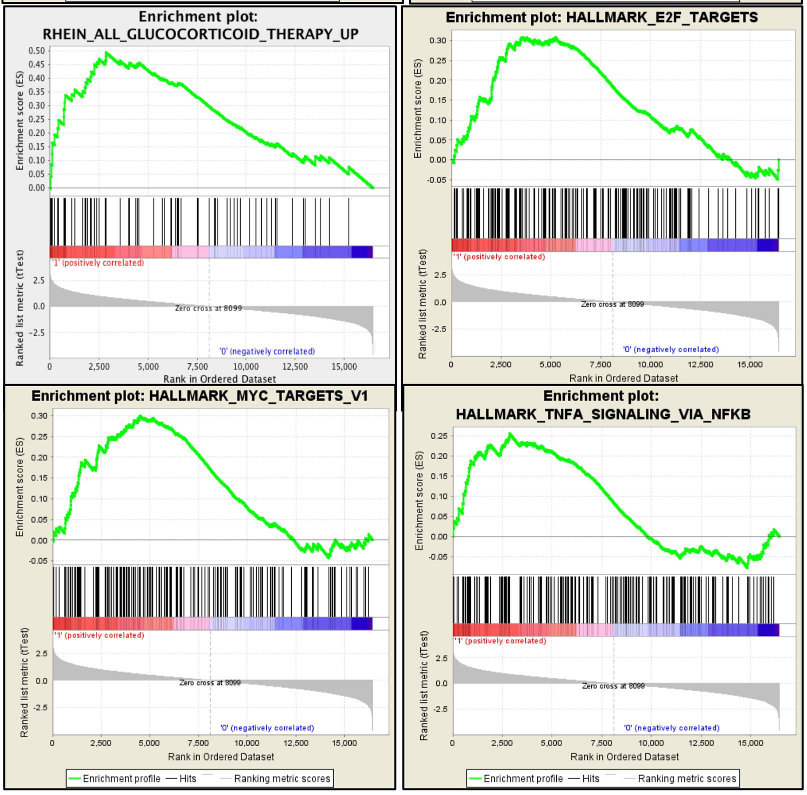

Figure S3: Phospho-kinase array: Proteome Profiler Human Phospho-Kinase Array (ARY003B) Kits from R&D Systems was used to detect relative phosphorylation changes in UDP-glucose stimulated cells.

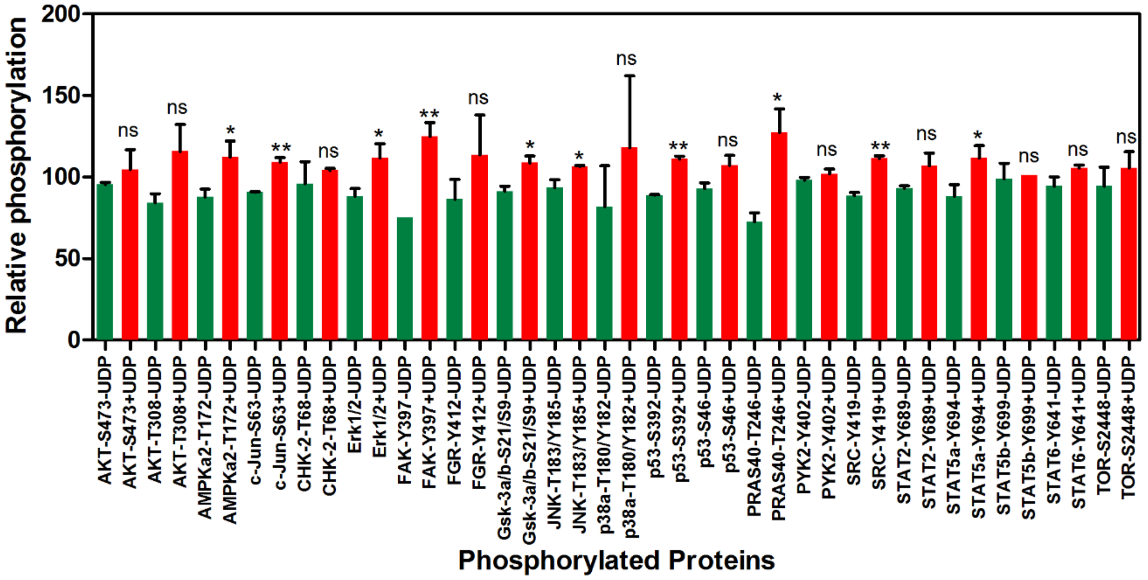

Figure S4: Stable transfection in Ba/F3 cells: Ba/F3 cells were stably transfected with the expression plasmid constructs pMSCVneo-empty vector (EV) and pMSCVneo-*P2RY14*-Myc-DKK (*P2RY14*) and were selected for 2 weeks using 0.8mg/ml G-418. The expression of *P2RY14* in the transfected cells was confirmed by Flow Cytometry (A) and Western Blot (B) using anti-FLAG antibodies.

A. **B.**

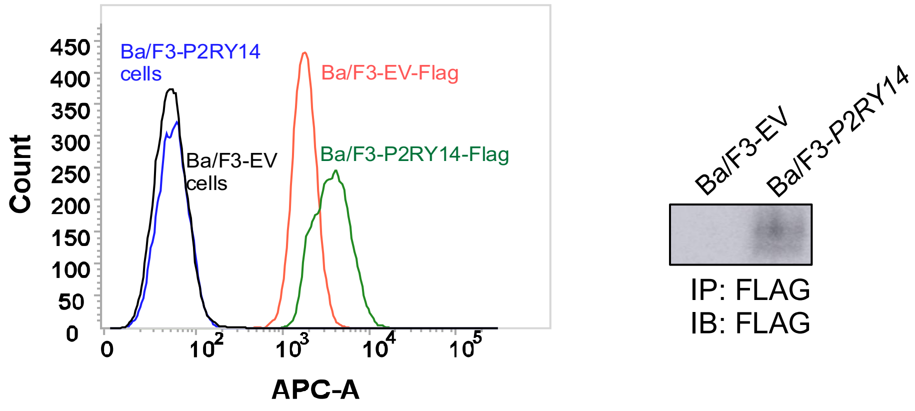

Figure S5: ERK phosphorylation decreased over the time after UDP-glucose stimulation: Ba/F3 cells stably transfected with *P2RY14* were stimulated with UDP-glucose. ERK1/2 phosphorylation was measured by quantification of bands after Western Blotting.

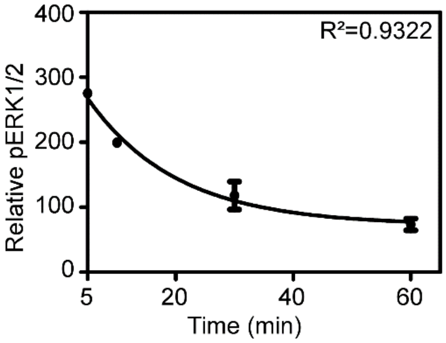

Supplement: Supplementary file 1 — Additional file includes supplementary materials and methods, supplementary tables S1-S2 and supplementary figures S1-S5. (PDF 997 kb) [file 13148_2018_516_MOESM1_ESM.pdf]
